# NVP-BEZ235 synergizes cisplatin sensitivity in osteosarcoma

**DOI:** 10.18632/oncotarget.23711

**Published:** 2017-12-27

**Authors:** Jin-Cheng Huang, Zhi-Fei Cui, Shui-Mu Chen, Lian-Jun Yang, Hong-Kai Lian, Bin Liu, Zhi-Hai Su, Jin-Shi Liu, Min Wang, Zheng-Bo Hu, Jia-Yao Ouyang, Qing-Chu Li, Hai Lu

**Affiliations:** ^1^ Department of Orthopedics, The Third Affiliated Hospital of Southern Medical University, Academy of Orthopedics, Guangdong Province, Guangzhou 510665, China; ^2^ Department of Orthopedics, Zhengzhou Central Hospital, Zhengzhou University, Zhengzhou 450000, China; ^3^ Department of Orthopedics, The Affiliated Shaoguan Hospital, Southern Medical University, Orthopedic Institute of Shaoguan, Shaoguan 512000, China

**Keywords:** osteosarcoma (OS), cisplatin, chemotherapy, autophagy

## Abstract

Osteosarcoma(OS) remains a major health concern in childhood and adolescence, although cisplatin is one of the gold standard chemotherapeutic drugs in the treatment of OS, chemoresistant to cisplatin is common. Phosphoinositide 3-kinase (PI3K)-Akt-mammalian target of rapamycin inhibitor (mTOR) pathway and autophagy regulates chemosensitivity incancer cells. In this study, we hypothesized that NVP-BEZ235, a dual inhibitor of PI3K/mTOR, could synergize cisplatin sensitivity in OS. *In vitro*, NVP-BEZ235 plus cisplatinexerted a synergistic effect on cell proliferation inhibition and apoptosis induction. Cisplatin could activate PI3K-Akt-mTOR pathway activity in early times, whereas, NVP-BEZ235 could inhibit PI3K-Akt -mTOR pathway activity all the times alone or combined with cisplatin. What's more, NVP-BEZ235 could switch function of autophagy induced by cisplatin to synergize cisplatin sensitivity. *In vivo*, pronounced decrease in tumor cell proliferation and increase in apoptosisin combination-treated mouse xenograft models compared with cisplatin or NVP-BEZ235 treated models. All these results suggest NVP-BEZ235 could synergize cisplatin sensitivity in OS, combination of NVP-BEZ235 with cisplatin could represent a novel therapeutic strategy for treatment of OS.

## INTRODUCTION

Osteosarcoma(OS) is a commonmalignant bone tumor predominantly seen in childhood and adolescence. Despite the combination of adjuvant chemotherapy and surgical removal of the tumor has improved the 5-year-survival rates dramatically, the 5-year-survival rates haven't improved in the past 30 years [1, 2]. To some extent, the underlying reason behind this may be chemoresistance. Although cisplatinbelongs to the gold standardMAP (methotrexate, cisplatin, doxorubicin) regimenin the treatment of OS [2, 3], high risk of cisplatin-associated nephrotoxcitiy, ototoxicity and gonadal dysfunction limit its individual doses [4], what's more, lots of patients are resistant to cisplatin. Thus, development of novel treatment strategies for enhancing cisplatin sensitivity in OS is imperative.

Phosphoinositide 3-kinase (PI3K)-Akt-mammalian target of rapamycin inhibitor (mTOR) pathway takes part in lots of disparate cellular functions(cell growth, proliferation, autophagy, apoptosis, chemo-resistance)and has been considered as a promising drug target for cancer therapy [5, 6]. For the past few years, PI3K-Akt-mTOR pathway was shown to participate in osteosarcoma development and metastasis [7–9], however, its role on OS chemoresitanceis unclear.

Autophagy, an evolutionarily conserved process, mediates lysosomal degradation of cytoplasmic and cellular organelles, plays an important role in maintaining the homeostasis [10, 11]. It is generally thought that autophagy has four functions in cells confronted with different conditions: cytoprotective, cytotoxic, cytostatic and nonprotective [12]. Recently, researches showed that autophagy induced by MAP in OS could promote chemoresitance [13, 14] andautophagy targeting methods could be used to enhance chemosensitivity in cancer cells [15–18].

NVP-BEZ235, a dual PI3K/mTOR inhibitor, couldinhibit the activity of PI3K-Akt-mTOR pathwayand the proliferation of OS cancer cells effectively [19, 20]. Moreover, in some cisplatin-resistant tumor cell lines, NVP-BEZ235 could enhance their cisplatin sensitivity [21–23]. However, whether NVP-BEZ235 could synergize cisplatin sensitivity or not in OS is still unknown.

In this study, we demonstrated that NVP-BEZ235 could synergize cisplatin sensitivity in OS both *in vitro* and *in vivo*. Our results suggest that there is value in utilizing NVP-BEZ235 to sensitize OS to cisplatin.

## RESULTS

### NVP-BEZ235 synergistically enhances the anti-proliferative effect of cisplatin on OS cells

**Figure 1:**
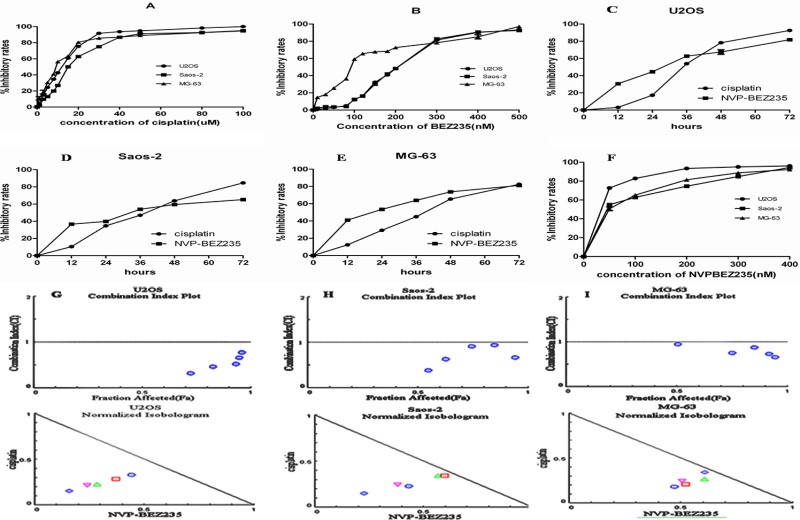
Effect of cisplatin or/and NVP-BEZ235 on OS U2OS, Saos-2 and MG-63 cells proliferation Cell viability was detected by CCK-8 assay. The results were expressed as inhibitory rates of cells. (**A–E**) Inhibitory rates in U2OS, Saos-2 and MG-63 cells after treatment with different doses of cisplatin or/and NVP-BEZ235. (**F**) Inhibitory rates in U2OS, Saos-2 and MG-63 cells after treatment with a 20000:1 fixed ratio combination of NVP-BEZ235 and cisplatin. (**G–I**) Combination Index plot and Isobologram for combination of NVP-BEZ235 and cisplatin in U2OS, Saos-2 and MG-63 cells. The CI plot for the combinations of drugs where synergy (identified by a Combination Index < 1) over a range of drug concentrations. Combination index (CI) value was calculated using CompuSyn software. The line represents an additive affect, where CI = 1. Each data point represents the mean ± SD of triplicate experiments.

First, effect of NVP-BEZ235 or/and cisplatin on the proliferation of OS U2OS, Saos-2 and MG-63 cells was determined by CCK-8 assay. As shown in Figure 1A–1E, NVP-BEZ235 or cisplatinalone could inhibit the proliferations of U2OS, Saos-2 and MG-63 cells in dose- and time-dependent manners. The 50% inhibitory concentration (IC50) value of cisplatinand NVP-BEZ235 were shown in Table 1. Second, to test the synergistic effect between cisplatin and NVP-BEZ235, U2OS, Saos-2 and MG-63 cells were exposed to increasing doses of NVP-BEZ235 or cisplatin alone or in a 20000:1 fixed ratio combination and the anti-proliferative effectwas assessed by CCK-8 assay (Figure 1F). Third, drug synergy was measured by fraction Combination indexplot and isobologram (Figure 1G–1I).

**Table 1:** The inhibitory potentials of cisplatin and NVP-BEZ235 on the viability of U2OS, Saos-2 and MG-63 cells

cells	Compound/IC50	
	Cisplatin (uM)	NVP-BEZ235 (nM)
U2OS	10.793 ± 0.097	198.433 ± 0.981
Saos-2	15.643 ± 0.193	197.800 ± 1.778
MG-63	7.873 ± 0.161	88.03 ± 0.531

As shown in Figure 1G–1I, both fraction Combination indexplot and isobologram showed that combination of NVP-BEZ235 with cisplatin exerteda synergistic anti-proliferative effecton OS U2OS, Saos-2 and MG-63 cells.

### Different regulation of PI3K-Akt-mTOR pathway activity in U2OS and Saos-2 cells by cisplatin and NVP-BEZ235

**Figure 2:**
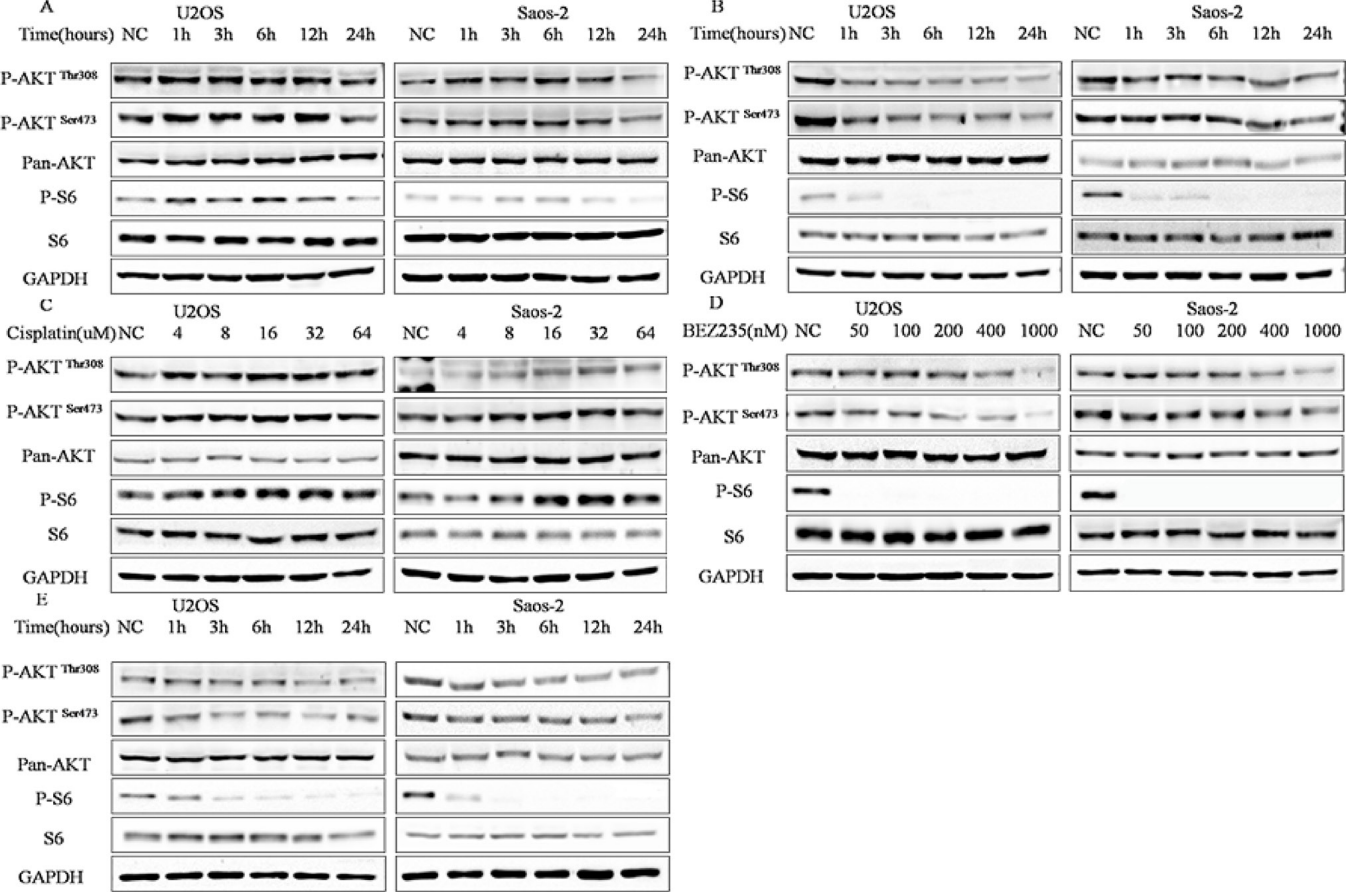
Different regulation of PI3K-Akt-mTOR pathway activity by cisplatin and NVP-BEZ235 in U2OS and Saos-2 cells (**A**) Kinetic effect of 4 uM or 6 uM cisplatin in U2OS and Saos-2 cells. (**B**) Kinetic effect of 200 nM NVP-BEZ235 on U2OS and Saos-2 cells. (**C**) Dose-ranging experiment 6 to 64 uM cisplatin in U2OS and Saos-2 cells treated for 12 h. (**D**) Dose-ranging experiment from 50 to 1000 nM NVP-BEZ235 in U2OS and Saos-2 cells for 12 h. (**E**) Kinetic effect of NVP-BEZ235 plus cisplatin treatment in U2OS and Saos-2 cells. GAPDH were used as loading controls.

As PI3K-Akt-mTOR pathway has dual effect on chemosensitivity in cancer cells [24–26], activity of PI3K-Akt-mTOR pathway in response to cisplatin or/and NVP-BEZ235 in U2OS and Saos-2 cellscells were analyzed. As shown in Figure 2, transient increase in phosphorylated-S6 and phosphorylated-Akt (Ser473, Thr308) were seen at 1–12 hours after treatment with cisplatin, while, all phosphorylation levels were decreased at 24 hours (Figure 2A). In contrast, phosphorylated-S6 and phosphorylated-Akt (Ser473, Thr308) levels decreased at all times (1–24 hours) after treatment with NVP-BEZ235 in U2OS and Saos-2 cells (Figure 2B). We further explored cisplatin-induced transient increase in PI3K-Akt-mTOR pathway activity and found a concentration dependent increase in PI3K-Akt-mTOR pathway activity at 12 hours in U2OS and Saos-2 cells (Figure 2C). While, a concentration dependent decrease in PI3K-Akt-mTOR pathway activity was found at 12 hours in U2OS and Saos-2 cells treated with NVP-BEZ235 (Figure 2D). As expected, NVP-BEZ235 plus cisplatin treatment decreased the PI3K-Akt-mTOR pathway activity at all times (1–24 hours) in U2OS and Saos-2 cells (Figure 2E).

Collectively, these results demonstrate that NVP-BEZ235 alone or combined with cisplatin could inhibit PI3K-Akt-mTOR pathway activity effectively, whereas, cisplatin could increase activity of PI3K-Akt-mTOR pathway in early times.

### NVP-BEZ235 synergistically induces apoptosis in U2OS and Saos-2 cells treated with cisplatin

**Figure 3:**
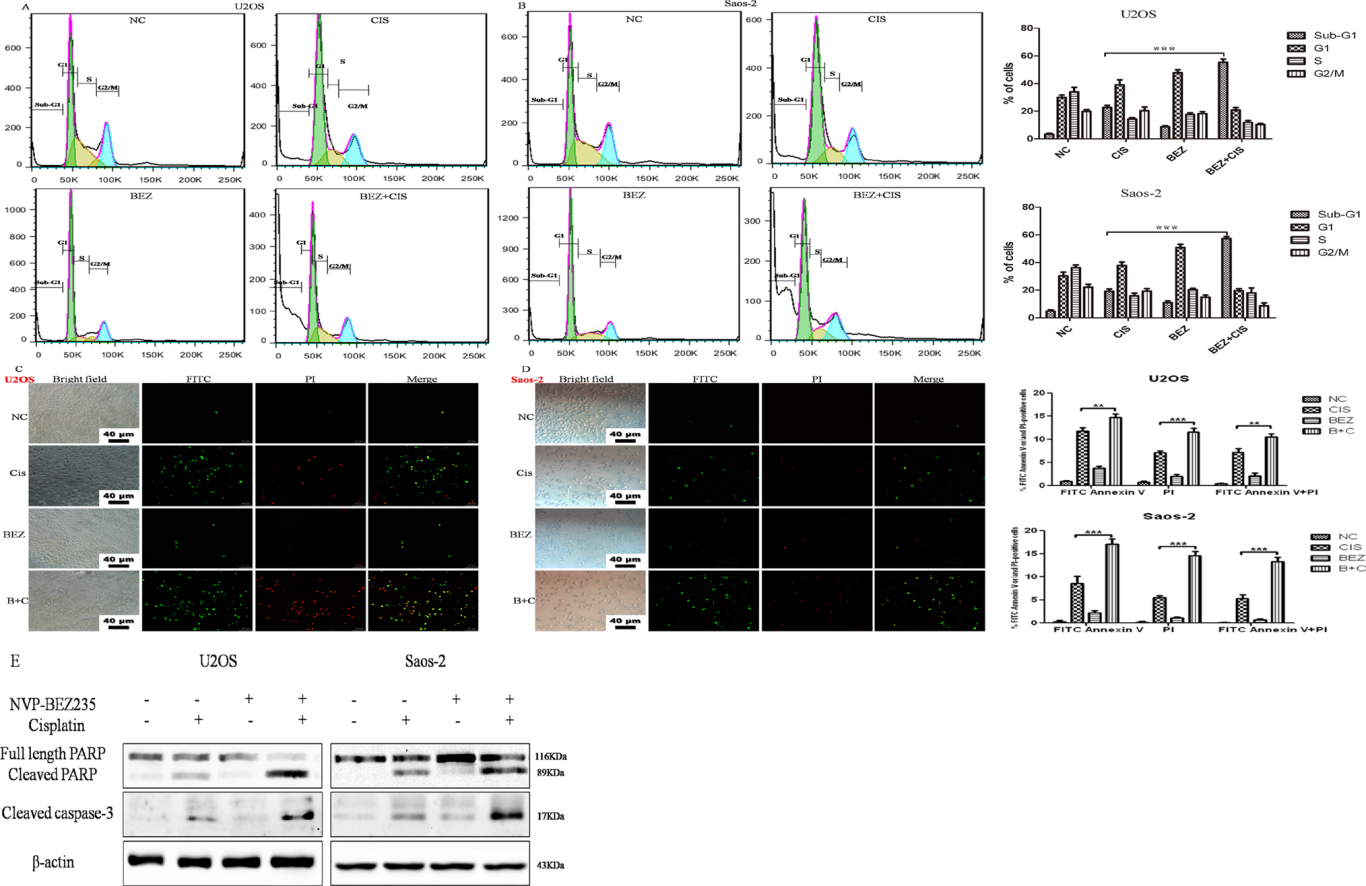
Effect of cisplatin or/and NVP-BEZ235 on U2OS and Saos-2 cells cell cycle progression and apoptosis (**A, B**) Summary of cell cycle progression after the treatment with cisplatin or/and NVP-BEZ235 in U2OS and Saos-2 cells for 24 hours. Data is shown as the % of cells in Sub-G1, G1, S and G2-M. (**C, D**) Representative results of Annexin V-PI dual staining with flow cytometry or fluorescence microscopy in U2OS and Saos-2 cells at 24 hours after treatment with cisplatin or/and NVP-BEZ235. (**E**) Cleaved PARP and cleaved caspase-3 were detected by Western blot in U2OS and Saos-2 cells at 24 hours after treatment with cisplatin or/and NVP-BEZ235. β-actin was used as a loading control. Each data represents the mean ± SD of triplicate experiments. Notes: Green: stained with Annexin V-FITC; red: stained with PI; Merge: stained with both Annexin V-FITC and PI. Apoptotic cells were highlighted by FITC, PI, and their merged images. ^**^P < 0.01 and ^***^P < 0.001. Scale bar = 40 μm. Cis is short for cisplatin, BEZ is short for NVP-BEZ235, B + C is short for combined NVP-BEZ235 and cisplatin.

To better understand the mechanism underlying the combined anti-proliferative activity observed in the CCK-8 assays, cell cycle progression were investigated in U2OS and Saos-2 cellsafter treatment with NVP-BEZ235 or/and cisplatin for 24 hours. As shown in Figure 3A and 3B, both NVP-BEZ235 and cisplatin single-agent treatment caused significant changes in cell cycle distribution, what's more, concomitant treatment resulted in a marked increase of cells in the sub-G1 phase compared with the untreated control, NVP-BEZ235, or cisplatinsingle-treated cells.

Next, the effect of NVP-BEZ235 or/and cisplatin on apoptosis was determined. U2OS and Saos-2 cells were exposed to NVP-BEZ235 or/and cisplatin for 24 hours, then stained with Annexin V-FITC and PI and analyzed by flow cytometry. As shown in Figure 3A, combined treatment with NVP-BEZ235 and cisplatin in U2OS and Saos-2 cells for 24 hours induced more apoptosis rates than treated with NVP-BEZ235 or cisplatin alone (*P* < 0.05). Also, fluorescence microscopy examination revealed similar results (Figure 3B). In addition, cleaved PARP and cleaved caspase-3 were significantly elevated compared to the cisplatin or NVP-BEZ235 groups (Figure 3C).

All these suggest that NVP-BEZ235 synergistically induces apoptosis in U2OS and Saos-2 cells treated with cisplatin.

### The synergistic effects of combined NVP-BEZ235 and cisplatin weren't due to enhanced activation of the p53 or TAp73 pathway

Tumor suppressor p53, a transcription factor, can be activated by DNA damage to regulate a list of target genes controlling cell cycle arrest, apoptosis and autophagy [27]. TAp73, one member of the p53 family, acts activities familiar with those of p53 [28]. Previous studies have shown that inhibition of PI3K-Akt-mTOR pathway activity could increase cellular levels of p53 [29, 30] and enhance DNA damage-induced cell death in tumor cells through a p53-dependent manner [24, 31, 32]. Also, inhibition of mTOR by rapamycin can up-regulate TAp73 expression [33] and synergize cisplatin sensitivity in basal-like breast cancer cells through a TAp73-dependent manner [34]. Our previous studies also showed that TAp73 could be reactivated in p53-mutant or p53-null cancer cells to exert activities familiar with those of p53 to suppress tumor [35–37].

**Figure 4:**
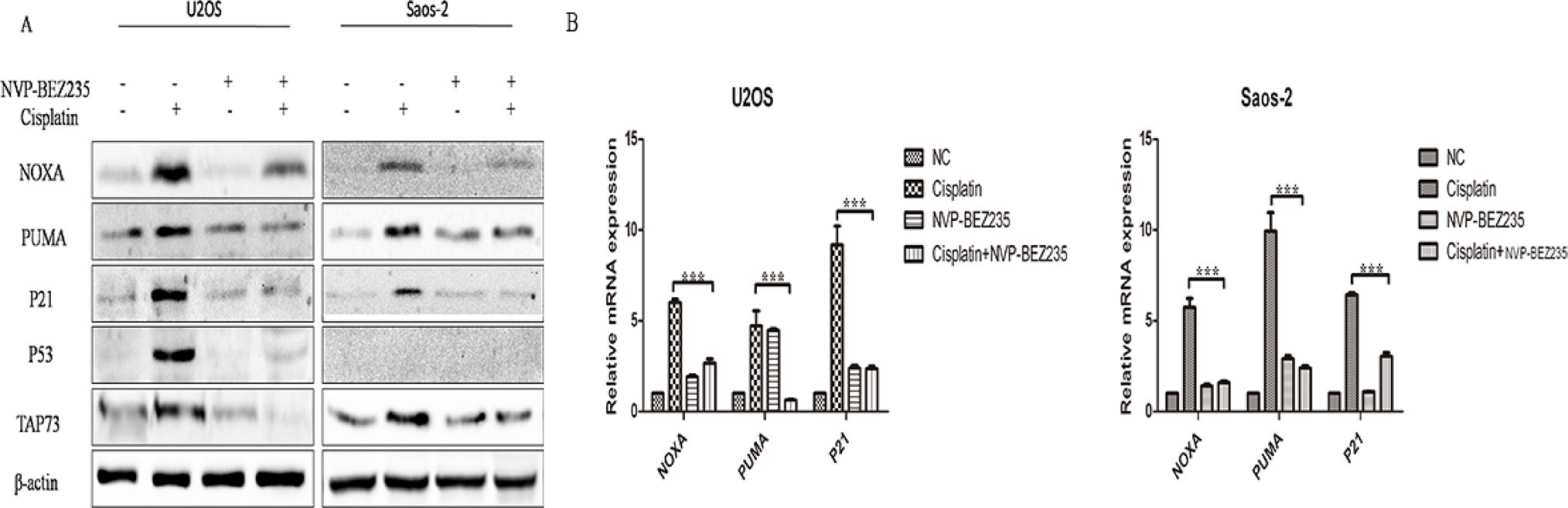
NVP-BEZ235 inhibits p53- and TAp73-dependent transactivation in U2OS and Saos-2 cells U2OS and Saos-2 cells were treated with cisplatin or/and NVP-BEZ235 for 24 hours. (**A**) Western blot was performed to detect p53, TAp73, NOXA, PUMA and P21 expression. β-actin was used as a loading control. (**B**) qPCR was performed to detect NOXA, PUMA and P21 at the mRNA levels. β-actin was used as a loading control. Each data represents the mean ± SD of triplicate experiments.

So, we decided to determine whether the synergistic effects of combined NVP-BEZ235 and cisplatin could be explained by enhanced activation of p53 or TAp73 pathway in U2OS and Saos-2 cells. Surprisingly, expression of p53 and TAp73 were down-regulated in combined NVP-BEZ235 and cisplatin treatment groups than cisplatin treatment groups (Figure 4A). What's more, levels of downstream transcriptional targets of p53 and TAp73 including NOXA, p21 and PUMA were also decreased at both protein and mRNA level (Figure 4B).

So, the synergistic effects of the combined NVP-BEZ235 and cisplatin weren't due to enhanced activation of the p53 or TAp73 pathway.

### NVP-BEZ235 sensitizes U2OS and Saos-2 cells to cisplatin through switching function of autophagy induced by cisplatin

Autophagy, an evolutionarily conserved process, mediates lysosomal degradation of cytoplasmic and cellular organelles, plays an important role in maintaining the homeostasis [10, 11]. It is generally thought that autophagy has tumor promotion or tumor suppression roles in different contexts [11, 12, 38]. Previous studies have demonstrated that autophagy induced by cisplatin promotes chemoresitance in OS [39, 40], and NVP-BEZ235 could enhance cisplatin sensitivity in some cisplatin-resistant cancer cells through autophagyinducing [21–23].

**Figure 5:**
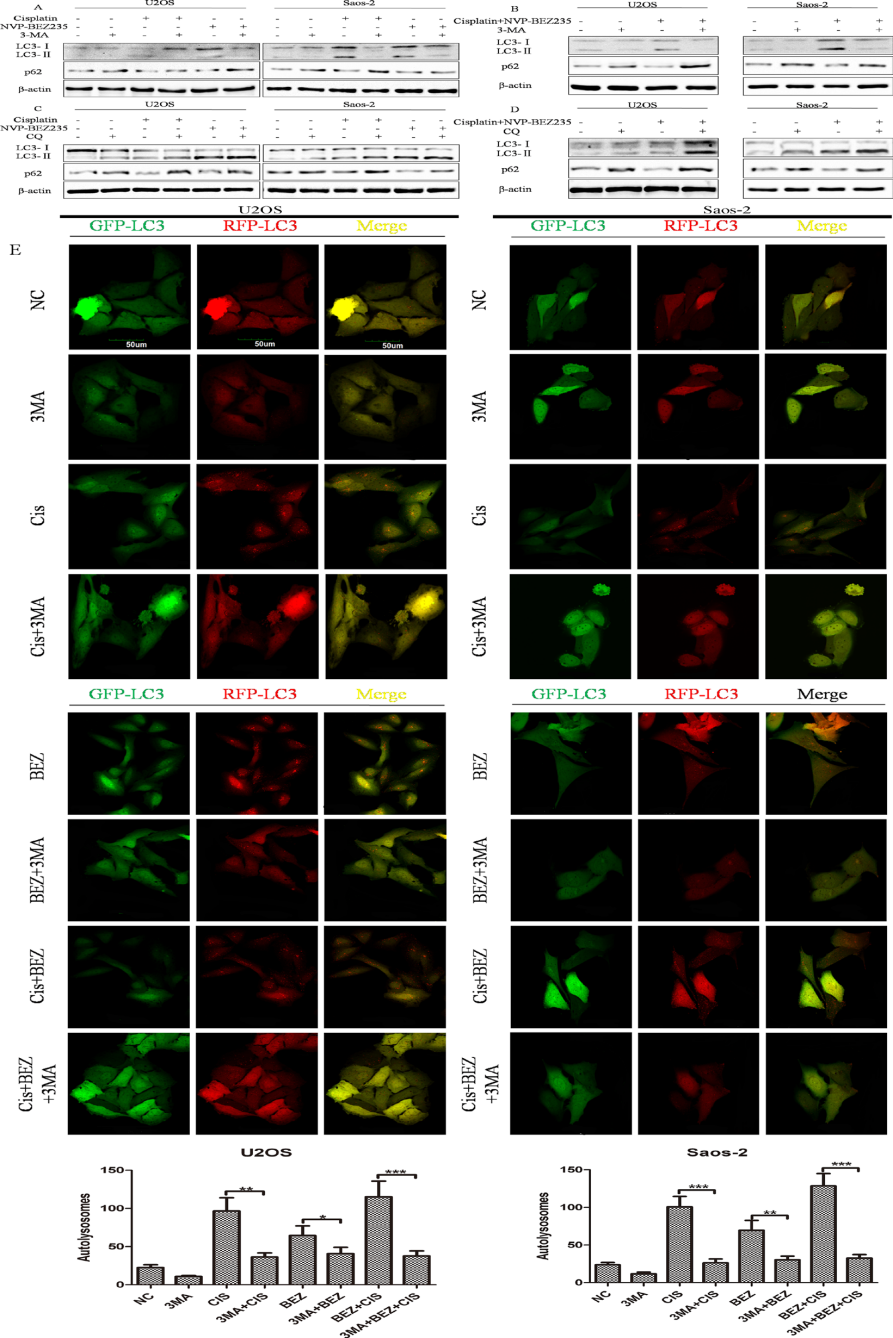
Cisplatin or/and NVP-BEZ235 treatment induce autophagy in U2OS and Saos-2 cells (**A–D**) LC3 and p62 expressions were detected by Western blot in U2OS and Saos-2 cells after treatment with cisplatin or/and NVP-BEZ235 with or without 3-MA or CQ. β-actin was used as the loading control. (**E**) Autophagy flux was induced by cisplatin or/and NVP-BEZ235 and inhibited by 3-MA. U2OS and Saos-2 cells were infected with GFP-RFP-LC3 adenovirus for 36 h and then treated with 24 μM cisplatin or/and 200 nM NVP-BEZ235 for another 24 h or pre-treated with 3 mM 3-MA for 3 h. The GFP-RFP-LC3 fluorescence was observed by a confocal microscope. Number of autolysosomes(red vesicles minus green vesicles) were calculated in per cell (*n* = 5). Data are shown as mean ± SD. ^*^P < 0.05, ^**^P < 0.01, ^***^P < 0.001. Scale bar = 50 um. Cis is short for cisplatin, BEZ is short for NVP-BEZ235.

In order to explore whether autophagy takes part in the synergistic effects of combined NVP-BEZ235 and cisplatin, functions of autophagy induced by cisplatin or/and NVP-BEZ235 in U2OS and Saos-2 cells were tested. As expected, cisplatin or/and NVP-BEZ235 treatment could induce autophagy in U2OS and Saos-2 cells, while 3-Methyladenine (3-MA) and Chloroquine Phosphate (CQ) could inhibit autophagy induced by cisplatin or/and NVP-BEZ235 effectively (Figure 5A–5E).

**Figure 6:**
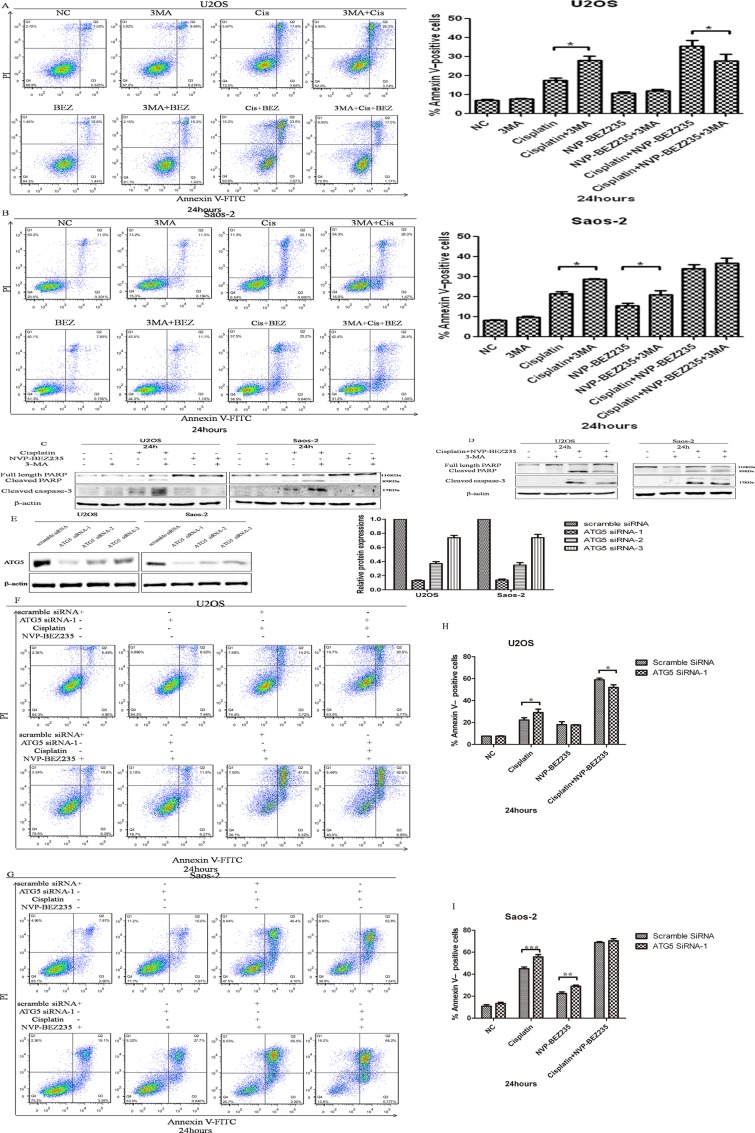
NVP-BEZ235 switches function of autophagy induced by cisplatin in U2OS and Saos-2 cells (**A, B**) Representative results of Annexin V-PI dual staining with flow cytometry in U2OS and Saos-2 cells treated with cisplatin or/and NVP-BEZ235 with or without 3-MA. (**C, D**) Cleaved PARP and cleaved caspase-3 expressions were detected in U2OS and Saos-2 cells treated with cisplatin or/and NVP-BEZ235 with or without 3-MA. (**E**) Generation of ATG5-deficient U2OS and Saos-2 cells with ATG5 siRNAs. (**F, G**) Representative results of Annexin V-PI dual staining with flow cytometry in U2OS and Saos-2 cells with or without ATG-5 siRNA-1 silence treated with combined cisplatin and NVP-BEZ235. (**H, I**) Cleaved PARP expressions were detected in U2OS and Saos-2 cells transfected with control or Atg5 siRNA treated with cisplatin or/and NVP-BEZ235. β-actin was used as the loading control. Each data represents the mean ± SD of triplicate experiments. ^*^P < 0.05, ^**^P < 0.01, ^***^P < 0.001.

Next, functions of autophagy induced by cisplatin or/and NVP-BEZ235 in U2OS and Saos-2 cellscells were analyzed by flow cytometry after exposed to 3-MA, cisplatin, NVP-BEZ235, singly or in combination. As shown in Figure 6A, 6B, percentage of apoptotic cells in combined cisplatin and 3-MA groups were higer than cisplatin groups in both U2OS and Saos-2 cells. In U2OS cells, apoptosis rates in combined 3-MA, cisplatin and NVP-BEZ235 groups were lower than combined cisplatin and NVP-BEZ235 groups. However, in Saos-2 cells, differences of apoptosis rates among combined cisplatin and NVP-BEZ235 groups and combined 3-MA, cisplatin and NVP-BEZ235 groups weren't significant. Similar results were obtained through detecting expressions of cleaved PARP by Western blot (Figure 6C, 6D).

In order to exclude the possible off-target effects by 3-MA, siRNA method wasapplied to selectively knockdown autophagy protein ATG5. As shown in Figure 6E, ATG5 siRNA-1and siRNA-2 could decrease expression of ATG5 effectivelyin both U2OS and Saos-2 cells. As expected, combined cisplatin and NVP-BEZ235 treatment induced lower apoptosis rates with ATG5 siRNA-1 silence in U2OS cells. However, differences of apoptosis rates in Saos-2 cells with or without ATG5 siRNA-1 silence treated with combined cisplatin and NVP-BEZ235 weren't significant (Figure 6F–6I). Similar results were obtained with ATG5 siRNA-2 silence.

Together, all these results show that NVP-BEZ235 sensitizes U2OS and Saos-2 cells to cisplatin through switching function of autophagy induced by cisplatin

### NVP-BEZ235 sensitizes OS cancer cells to cisplatin *in vivo*

To detect the *in vivo* efficacy of combined treatment of NVP-BEZ235 plus cisplatin, U2OS xenograft mice models were established and divided into 4 groups: (1) mice treated with vehicle only, (2) mice treated with cisplatin only, (3) mice treated with NVP-BEZ235 only, (4) mice treated with combined cisplatin and NVP-BEZ235.

**Figure 7:**
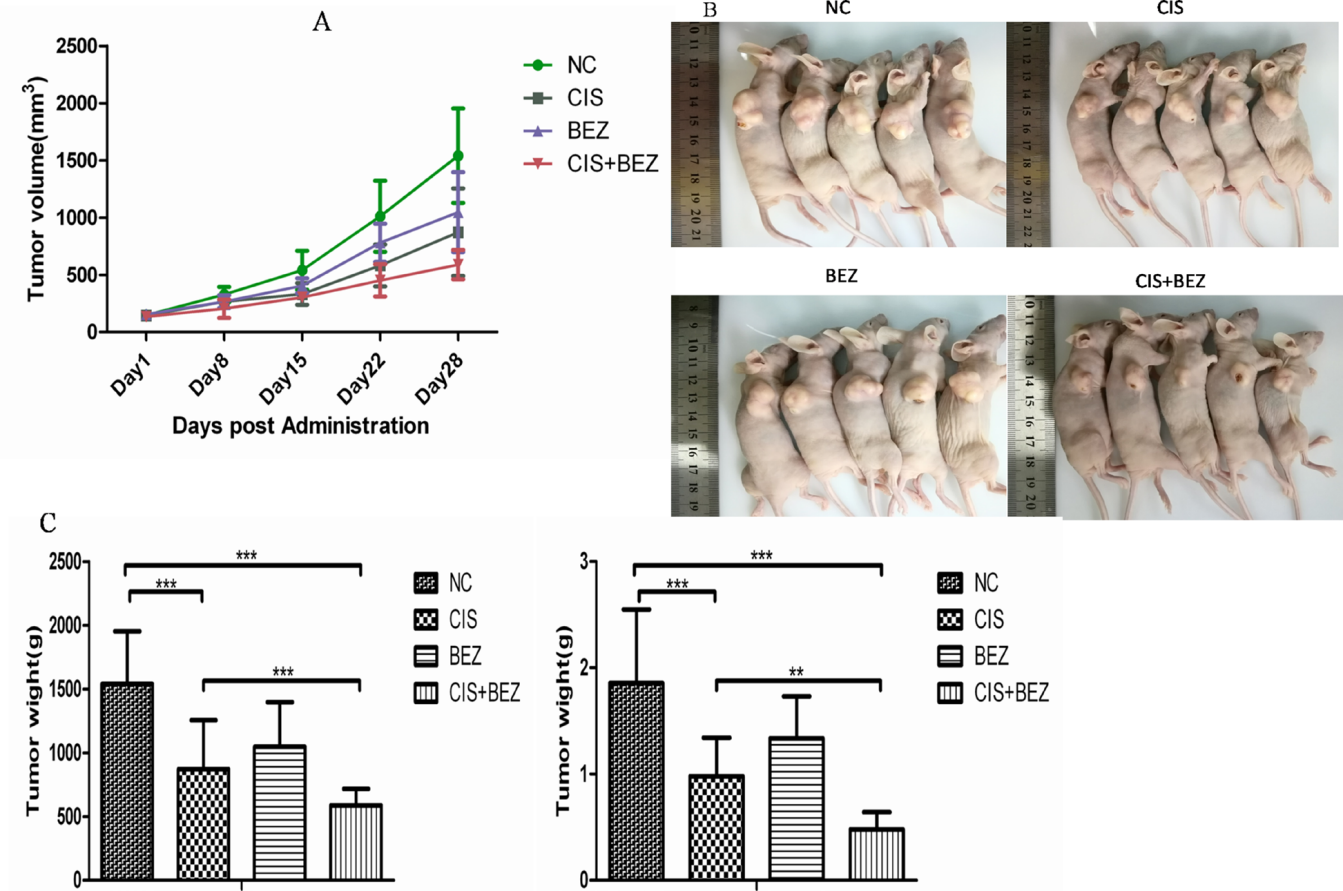
NVP-BEZ235 synergistically enhances growth inhibition effect of cisplatin *in vivo* (**A**) Tumor volume of each group was calculated by a caliper every 7 days. (**B, C**) Tumor volume and weight from each group on the 5th week after tumor implantation are shown. Data are presented as mean tumor volume ± SE. ^*^P < 0.05, ^***^P < 0.001.CIS is short for cisplatin, BEZ is short for NVP-BEZ235.

Tumors were allowed to develop for 35 days after injection, and therapy was started on day 7 after tumor implantation. The volume of the tumors was measured weekly until the mice were sacrificed. As shown in Figure 7A, from day 15, there were significant differences in tumor volume between combined NVP-BEZ235 and cisplatin groups and cisplatinsingle treatment groups. On day 28, all mice were sacrificed and tumor volumes were measured. Consistent with above results, tumor volume in combined NVP-BEZ235 and cisplatin groups were lesser than mice treated with vehicle, cisplatin or NVP-BEZ235 alone (Figure 7B–7C). This indicates that NVP-BEZ235 could synergistically enhance growth inhibition effect of cisplatin *in vivo*.

**Figure 8:**
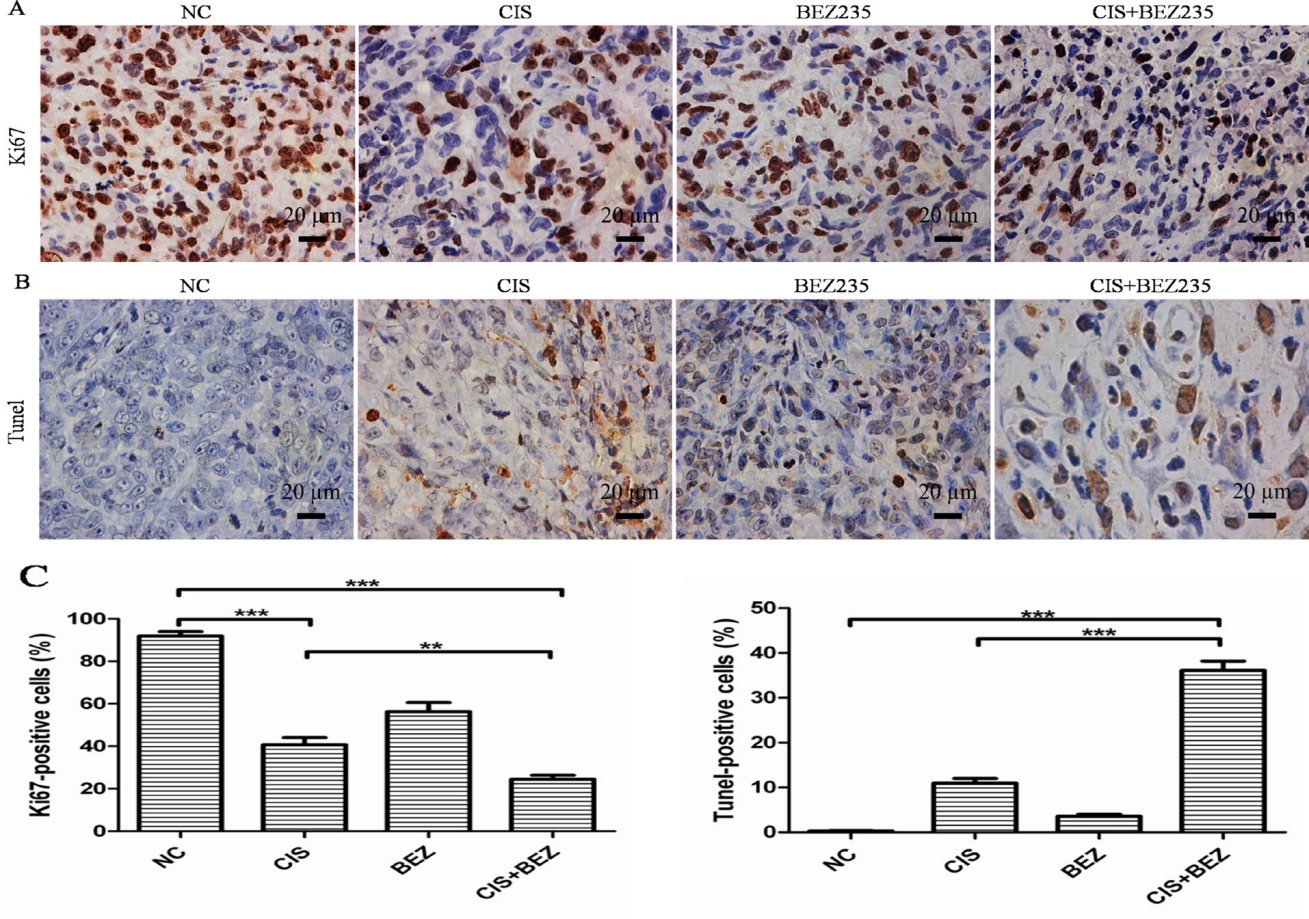
Combined NVP-BEZ235 and cisplatin increases cell apoptosis and decreases cell proliferation compared with cisplatin treatment alone *in vivo* (**A**) Cell proliferation was evaluated by Ki67 staining in tumor tissue (Scale bar = 20 um, magnification, 400×); (**B**) Apoptotic cells were labeled by TUNEL assay (Scale bar = 20 um, magnification, 400×); (**C**) Quantification of Ki67-positive and TUNEL-positive cells in each group (mean ± S.D., *n* = 5). ^**^P < 0.01, ^***^P < 0.001. CIS is short for cisplatin, BEZ is short for NVP-BEZ235.

Then, a pronounced decrease in tumor cell proliferation (Ki67) (Figure 8A, 8C) and increase in apoptosis (TUNEL-positive tumor cells) were also noted in combination-treated xenografts based on immunostaining (Figure 8B). Taken together, these data recapitulate the observations made *in vitro* and demonstrate that NVP-BEZ235 displays synergistic activity with cisplatin *in vivo* xenograft models.

## DISCUSSION

Although the underlying mechanisms of chemoresistancein OS have always been the focus of researchers all over the world [2, 41], identification of novel strategies to circumvent chemoresitance are still desperately required. For the past few years, PI3K-Akt-mTOR pathway has been demonstrated to participate in OS development and metastasis [7–9], however, its role on OS chemoresitanceis unclear.In this study, we demonstrated that NVP-BEZ235, a dual PI3K/mTOR inhibitor, could synergize cisplatin sensitivity in OS through switching function of autophagy induced by cisplatin. To our knowledge, our work is the first study trying to enhance cisplatin sensitivity in OS through switching function of autophagy by inhibition of PI3K-Akt-mTOR pathway activity.

In this study, we first detected the proliferation of OS U2OS, Saos-2 and MG-63 cellsafter treatment with cisplatin or/and NVP-BEZ235. Results showed that NVP-BEZ235 could synergistically enhance the anti-proliferative effect of cisplatin on OS cells. Then, cell cycle progression and apoptosis rates were analyzed. As shown in Figure 3, NVP-BEZ235 could synergistically induce apoptosis in U2OS and Saos-2 cells treated with cisplatin. All these indicate there may be possible in utilizing NVP-BEZ235 to sensitize OS to cisplatin.

As PI3K-Akt-mTOR pathway is always activated in human cancers, it is believed to be the target for inactivation in order to achieve better chemotherapy [42]. Consistent with otherstudies [21–23, 43, 44], in this study, cisplatin couldtransientlyincrease the activity of PI3K-Akt-mTOR pathwaywhereasNVP-BEZ235 alone or combined with cisplatin could inhibit PI3K-Akt-mTOR pathway activity effectively. So, it seems NVP-BEZ235 may enhance cisplatin sensitivity in OS through PI3K-Akt-mTOR pathway inactivation.

Tumor suppressor p53, the guardian of the genome, plays important roles in regulating chemosensitivity in cancer cells [27]. TAp73, one member of the p53 family, acts activities familiar with those of p53 [28, 45]. It's reported that inhibition of PI3K-Akt-mTOR signaling could increase cellular levels of p53, and augment p53 transcriptional activity [24, 29–32]. Similarly, mTOR inhibition also increases TAp73 expression [33] and synergizes cisplatin sensitivity in basal-like breast cancer cellsthrough a TAp73-dependent manner [34]. However, in contrast to above findings, J Bar *et al.* reported that exposure to LY294002(PI3K inhibitor), could abort the activation of p53 by drugs used in cancer chemotherapy [46]. Kojima, K *et al.* demonstrated that PI-103 (dual PI3K/mTOR inhibitor), could inhibit expression of p53 and p53-related pro-apoptotic proteins (p21, puma, noxa) [47]. R Suvasini *et al.* found that LY294002 could inhibit p53 activation on DNA damage [48]. So, we first detect whether the synergistic effects of combined NVP-BEZ235 and cisplatin could be explained by enhanced activation of p53 or TAp73 pathway in U2OS and Saos-2 cells. However, to our surprise, combined NVP-BEZ235 and cisplatin treatment decreased cisplatin-induced p53 and TAp73 and their targeted gene (*noxa, puma, p21*) expressions. As a result, it seems that the synergistic effects of combined NVP-BEZ235 and cisplatin aren'tdue to enhanced activation of p53 or TAp73 pathway in OS, PI3K-Akt-mTOR pathway regulates p53 or TAp73 pathway in a cell type dependent manner.

Autophagy, an evolutionarily conserved process, mediates lysosomal degradation of cytoplasmic and cellular organelles, has different functions in cells: cytoprotective, cytotoxic, cytostatic and nonprotective [12]. Researches demonstrated that p53 status [18, 49], immunogenic signal [15, 16], apoptosis ability [50], HMGB1 redox status [51] can determine the role of autophagy, and autophagy targeting methods can be used to enhance chemosensitivity in cancer cells [52]. As PI3K-Akt-mTOR negatively regulates autophagy, function of autophagy induced by cisplatin or/and NVP-BEZ235 in U2OS and Saos-2 cells were tested. Consistent with others’ results [14, 39, 40], autophagy induced by cisplatin promotes chemoresitance, however, combined NVP-BEZ235 and cisplatin treatment switched cytoprotective autophagy induced by cisplatin to cytotoxic(in U2OS cells) or nonprotective (in Saos-2 cells). So, it seems that NVP-BEZ235 could sensitize OS to cisplatin through switching autophagy function. However, the underlying mechanism how NVP-BEZ235 switches function of autophagy deserves our further exploration.

Although *in vitro* data showed that NVP-BEZ235 could be used to enhance cisplatin sensitivity in OS cancer cells, these results must be confirmed and validated *in vivo*. In our study, we extended *in vivo* experiments and demonstrated that cisplatin combined with NVP-BEZ235 induced more tumor growth delay compared to cisplatin or NVP-BEZ235 treatment alone. What's more, a pronounced decrease in tumor cell proliferation (Ki67) and increase in apoptosis (TUNEL-positive tumor cells) were noted in combination-treated xenografts based on immunostaining. All these results comfirmed that NVP-BEZ235 could synergize cisplatin sensitivity in OS both *in vitro* and *in vivo*.

NVP-BEZ235, an orally available dual PI3K/mTOR inhibitor, has been shown to inhibit PI3K-Akt-mTOR signal activity effectively and have antitumor activity [21–23]. Recently, its efficacy and safetywere assessed in a few Phase I/II clinical trials (www.clinicaltrials.gov). The results of our work showed that NVP-BEZ235 could synergize cisplatin sensitivity in OS and may be a promising adjuvant drug for OS. However, the underlying mechanism why NVP-BEZ235 switches function of autophagy induced by cisplatin deserves our future exploration.

## MATERIALS AND METHODS

### Cell lines and culture

The human osteosarcoma cell lines U2OS, Saos-2 and MG-63 cells were obtained from ATCC. U2OS and Saos-2 cells were cultured in McCoy's 5A medium (Gibco, 16600–082) supplemented with fetal bovine serum (corning, 35-076-CV) a final concentration of 10% for U2OS and 15% for Saos-2. MG-63 cells were maintained in Eagle's Minimum Essential Medium(ATCC, 30–2003) supplemented with 10% fetal bovine serum. The medium was replaced with fresh medium as necessary, and cultures were maintained at 37°C in the presence of 5% CO_2_.

### Chemical compounds and drugs

NVP-BEZ235 (S1009), cisplatin (S1166), 3-Methyladenine (S2767) and Chloroquine Phosphate (S4157) were obtained from Selleck Chemical in U.S.A. NVP-BEZ235 was provided as a 20 nM stock solution in 100% DMF. Final DMF concentration was kept constant at 0.1% in control and compound-treated cells. Cisplatin solution was provided as a 1 mg/ml stock solution in 0.9% isotonic saline solution. 3-Methyladenine solution was provided as a 100 mM stock solution in PBS. Chloroquine Phosphatesolution was provided as a 50 mM stock solution in water. Working solutions were prepared freshly before addition to the cell media.N-Methylpyrrolidine (NMP,120-94-5) and Polyethylene glycol 300 (PEG300,1546423) were bought from Sigma-Aldrich.

### Antibodies

The following antibodies were used for Western blotting : p73(abcam, USA); p53, Cleaved Caspase-3, PARP (ABclonal, USA); Puma, p21 Waf1/Cip1, S6 Ribosomal Protein, Phospho-S6 Ribosomal Protein, pan-Akt, Phospho-Akt (Ser473), Phospho-Akt(Thr308), NOXA, GAPDH, β-Actin(Cell Signaling Technologies, USA).

### Cell viability by CCK-8 assay

Cells were treated with cisplatin (1–100 μm) or/and NVP-BEZ235 (10 nM–500 nM) and the inhibitory effect on cell viabilitywas determined by CCK-8 assay (Dojindo Molecular Technologies, Japan) according to the manufacturer's instructions. In brief, cells were seeded into a 96-well plate (5 × 10^3^ cells/well) and cultured as described above. After treatment for 48 h, 10 μL of CCK-8 dye was added to each well, cells were incubated at 37°C for 2 h, then, optical density was read at 450 nm in a multi-mode microplate reader (Synergy2, BioTek, Winooski, VT). Inhibitory rates were calculated by Microsoft Excel and IC50 values were calculated using the Calcusyn software.

### Determination of synergism

For drug combination experiments, cells were treated with a 20:1 fixed ratio combination of NVP-BEZ235 and cisplatinfor 48 h. The data were analyzed by CompuSyn software with the results showed as fraction Combination indexplot and isobologram. According to the median-effect principle, where CI <1, =1, and >1 indicate synergism, additive effect, and antagonism, respectively.

### Western blot analysis

Cells were lysed with 2% SDS, 10% glycerol, 2 M urea, 10 mM Tris-HCl (pH 6.8), 10 mM dithiothreitol and 1 mM phenylmethylsulfonyl fluoride. The lysates were centrifuged and the supernatants were separated by SDS–polyacrylamide gel electrophoresis and blotted onto a nitrocellulose membrane (Bio-Rad Laboratories). The membrane was then incubated at 4°C overnight with the primary antibody, and subsequently incubated with the HRP conjugated secondary antibody at room temperature for 1 h. Immunoreactive proteins were detected the Western lightning Plus (PerkinElmer) according to the manufacturer's instructions and visualized using the GeneGnome XRQNPC system(SYNGENE). All protein bands were normalized against β-Actin protein. For result analysis, we first normalized the band intensity of the target protein to β-actin in each sample, correspondingly. We then normalized the relative target protein levels of the treatment groups to their control group.

### Cell cycle analysis

Cell cycle progressionwas detected using the Cell Cycle Detection Kit (Nanjing Keygen Biotech, China) according to the manufacturer's instructions. Briefly, cells were incubated with cisplatin or /and NVP-BEZ235for 24 h, trypsinized, washed and harvested, fixed in 70% ethanol and cell cycle analysis was done using propidium iodide. Samples were analyzed by FACScan flow cytometer (BD Biosciences, CA). The percentage of cells in different phases of the cell cycle was calculated using FlowJo 9.3.0 software (Tree Star Inc., Ashland, OR, USA).

### Real-time reverse transcriptase PCR

Isolated RNA was subjected to reverse transcription and PCR, as described previously [35]. The RNA isolater Total RNA Extraction Reagent(Vazyme, China) was used to isolate total RNA. Reverse transcription was performed using the HiScript II Q RT SuperMix for qPCR (Vazyme, China) to synthesize first-strand cDNA. SYBR green was used to detect dsDNA product during the real-time PCR reaction. The mRNA content was normalized to the housekeeping gene b-actin. The specific primer sequences used for real-time PCR were as follows: for NOXA forward, ATTACCGCTGGCCTACTGTG and reverse, ATGTGCTGAGTTGGCACTGA; PUMA forward, GGCCCAGACTGTGAATCC and reverse, TCACACG TGCTCTCTCTAAACC; P21 forward, TGGTGGCAGTA GAGGCTATG and reverse, AGTCCAGGCCAGTA TGTTAC.ACTIN forward, GCATGGGTCAGAAG GATTCCT and reverse, TCGTCCCAGTTGGTGACGAT.

### Detection of apoptosis *in vitro* and *in vivo*

*In vitro*, apoptosis was detected using the Annexin V-FITC Apoptosis Detection Kit (Nanjing Keygen Biotech, China) according to the manufacturer's instructions. In brief, the cells were harvested and washed twice with cold PBS, then resuspended in 500 μL binding buffer. Thereafter, 5 μL Annexin V-FITC and 5 μL PI were added and the cells were further incubated in the dark for 15 min at room temperature. Cells were then analyzed on a FACScan flow cytometer (BD Biosciences, CA). *In vivo*, DNA fragmentation using terminal deoxynucleotidyl transferase–mediated nick end labeling (TUNEL) was conducted to assess apoptosis in tumor tissue following the manufacturer's directions (Merck Corp. Cat. QIA39). The number of TUNEL-positive cells was calculated at 400× magnification for 5 fields randomly selected in each tumor sample.

### mRFP-GFP-LC3 expressing cells generation and fluorescent LC3 puncta analysis

To monitor the various stages of autophagy, the tandem GFP-RFP-LC3 adenovirus construct purchased from HanbioInc (Shanghai, China) was used in this study according to the manufacturer's instructions.In brief, to perform image-based analysis for autophagy, simply infected cells with the tandem GFP-RFP-LC3 adenovirus for 36 hours, thencells were treated and imaged for GFP and RFP by using confocal fluorescence microscopy. Green vesicles are considered to be autophagosomes and red vesicles are considered to be both autophagosomes and autolysosomes. The number of autolysosomes was achieved by subtracting the number of green vesicles from that of the red vesicles.

### siRNA knockdown

We transiently transfected cells with ATG5 siRNA using Lipofectamine RNAi MAX (Invitrogen, USA) in Opti-MEM medium (Invitrogen, USA), according to the manufacturer's instructions. The efficiency of transfection was measured by western blot. The siRNA sequences targeting ATG5 were:1) CATCTGAGCTACCCGGATA, 2) CATCTGAGCTACCCGGATA and 3) GCTAGCTGGC TGTCCATAT. The non-silencing sequence was AGGCTA TGAAGAGATAC.

### Mice U2OS xenograft

Female BALB/c mice aged 4 to 6 weeks were purchased from the animal facility of Southern Medical University of China. All mice were maintained in the accredited animal facility of Southern Medical University, and maintained in accordance with the guidelines of NIH. 5 × 10^7^ U2OS cells were injected subcutaneously into the right flank of the mice. When the right flank xenografts were established at about 150 mm^3^, animals (5 mice per group) were administrated with cisplatin (5 mg/kg in 0.9% isotonic saline solution through intraperitoneal injection), NVP-BEZ235 (45 mg/kg in 10% NMP–90% PEG300 through oral gavage) or combination. All drugs were freshly prepared and given twice a week for 28 consecutive days. Control mice received vehicle only. All mice were sacrificed 4 weeks after treatment. Tumor volumes (mm^3^) and weight were measured and tumors from each group were excised for histological study. All animals were maintained in accordance with the guidelines of NIH.

### Immunohistochemistry (IHC) analysis

Expression of Ki67 in xenograft tumor tissues were analyzed by IHC staining. Briefly, tissues were deparaffinized, rehydrated, and subjected to 5-min pressure-cooking antigen retrieval, 10-min double endogenous enzyme block, and overnight primary antibody incubation, and subjected to prediluted biotinylated pan-specific universal secondary antibody (Vector laboratories) for 10 min. Signals were detected by adding 3,3′-diaminobenzidine (DAB) substrate hydrogen peroxide and counterstained by hematoxylin QS. All reagents were obtained from Vector Laboratories (Burlingame, CA). Positive expression was defined as >15% positive staining in cell population.

### Statistical analysis

Data are expressed as mean ± standard deviation (S.D.). Every experiment was performed in triplicates and repeated two or three times. Statistical analysis was performed using SPSS 13.0 software. All data involving multiple groups were analyzed with one-way ANOVA followed by Dunnett's test. A value of *P* < 0.05 was considered statistically significant.

## Abbreviations

OS: Osteosarcoma
PI3K: Phosphoinositide 3-kinase
mTOR: mammalian target of rapamycin
3-MA: 3-Methyladenine
CQ: Chloroquine Phosphate.
